# Cardiac Symptoms During the Russia-Ukraine War: A Google Trends Analysis

**DOI:** 10.7759/cureus.36676

**Published:** 2023-03-25

**Authors:** Tsering Dolkar, Smitha Gowda, Saurav Chatterjee

**Affiliations:** 1 Internal Medicine, One Brooklyn Health (OBH) Interfaith Medical Center, Brooklyn, USA; 2 Internal Medicine, University of South Dakota, Sanford School of Medicine, Sioux Falls, USA; 3 Cardiology, Northwell Health/Long Island Jewish Hospital, New York, USA

**Keywords:** google trends, palpitations, dyspnea, chest pain, ukraine-russia war

## Abstract

Background

The 2022 Ukraine-Russian War has led to significant anxiety, anguish, and trauma among the people in Ukraine. The objective of this study was to analyze the Google Trend results of common cardiac symptoms in Ukraine, Russia, and worldwide in 2022 and compare that to 2021 with the hypothesis that common cardiac symptoms in the war-affected regions would be higher compared to the rest of the world. We hypothesize that the search trends of cardiac symptoms would increase in Ukraine given the turmoil caused by the Russian invasion.

Methods

We queried Google Trends for common cardiac symptoms such as chest pain, dizziness, palpitations, syncope, etc. Google Trends provides results as relative search volume (RSV) displayed in a geographical format. The RSV ranges from 0 to 100, with 0 indicating that the search term is not popular, and 100 indicating the search term’s popularity is at its peak. Google Trends of cardiac symptoms in Russia, Ukraine, and worldwide was taken two weeks before and after February 24, 2022, compared with the same period in 2021. To assess the difference in Google Trends between the study periods in 2022 and 2021, the paired t-test was used.

Results

Overall, Google Trends for cardiac symptoms was lower in Ukraine and Russia than worldwide, in both 2021 and 2022 during the study period. There was a significant reduction in search for chest pain (14 vs. 30.5; p<0.049), pedal edema (40.0 vs 66.6; p approaching 0), and syncope (37.8 vs. 58.4; p<0.002) in Ukraine during the study periods in 2022 compared to 2021. There was a decrease in the searches for dyspnea (44.6 vs. 55.4; p<0.029) in Russia and for dizziness (87.6 vs. 92.8; p<0.005) worldwide. There was an increase in the searches for edema (93.6 vs. 91; p <0.002) and for fatigue (88.6 vs 79.5; p approaching 0) worldwide in study periods in 2022 as compared to 2021. There was no other significant difference between cardiac symptom search trends during the periods evaluated in Ukraine, Russia, and worldwide.

Conclusion

There appears to be a significant reduction in searching for a few cardiovascular symptoms, namely, chest pain, pedal edema, and syncope in Ukraine, which may be due to a focus on other immediate problems related to war and the availability of the Internet.

## Introduction

Russia invaded Ukraine on February 24, 2022 [1]. Armed conflict not only increases communicable diseases but has been shown to increase coronary artery disease, cerebrovascular disease, blood pressure levels, tobacco, and alcohol use [2]. Search engine tools like Google are widely used as the first screening tool to gain more information regarding one’s symptoms before patients seek professional medical attention. People often use Google searches before seeking professional help [3]. Google Trends is a tool to analyze the popularity of search queries on Google on the web. It can provide a wide variety of search trends from different geographic regions at different periods. Google is a widely used search engine and Google Trends helps generate trends in Internet searches across the web. So, Google Trends can give clues into public health-seeking behavior. The objective of this study was to analyze the Google Trends results of common cardiac symptoms for Ukraine, Russia, and worldwide for 2022 and compare that to 2021 with the hypothesis that common cardiac symptoms in the war-affected regions would be higher compared to the rest of the world.

## Materials and methods

Common cardiac symptoms, such as chest pain, dizziness, palpitations, syncope, etc., were taken from Braunwald’s Cardiology 12th Edn. [4]. Google Trends was accessed at http://trend.google.com. The exact search terms used were chest pain, dizziness, palpitations, shortness of breath (the lay term for dyspnea), syncope, cough, heartburn, fatigue, leg swelling (the lay term for pedal edema), shoulder pain, and nausea. These layman's terms were used for dyspnea and pedal edema because they generated overall more results than dyspnea or pedal edema alone. The regions selected for each search term were Ukraine, Russia, and worldwide. The search period selected for each search term was February 10, 2022, to March 10, 2022, and February 10, 2021, to March 10, 2021.

Google Trends provides results as relative search volume (RSV) displayed in a geographical format. The RSV ranges from 0 to 100, with 0 indicating that the search is not popular and 100 indicating the term’s search popularity is at its peak. Instead of showing the absolute search volume, Google Trends shows the relative search volume, which is normalized. It basically compresses a keyword's maximum search countdown to 100.

Google Trends of cardiac symptoms in Russia, Ukraine, and worldwide was taken two weeks before and after February 24, 2022, and compared with the same period in 2021. To assess the difference in Google Trends between the study periods in 2022 and 2021, the paired t-test was used. A p-value of <0.05 was considered statistically significant. Ethical approval for the analysis was not required, as these queries are anonymous and not associated with any physical location.

## Results

**Figure 1:**
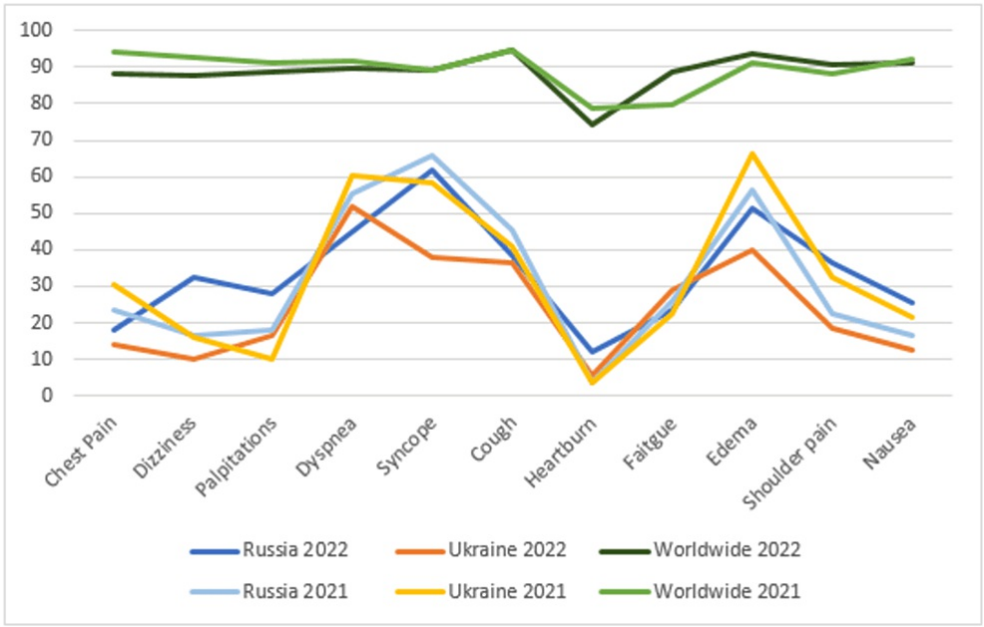
Comparing the means of cardiac symptoms in 2022 with those in 2021

Overall, Google Trends for cardiac symptoms were lower in Ukraine and Russia than worldwide in 2021 and 2022 during the study period (Figure 1).

There was a significant reduction in search for chest pain (14 vs. 30.5; p<0.049), pedal edema (40.0 vs 66.6; p approaching 0), and syncope (37.8 vs. 58.4; p<0.002) in Ukraine during the study periods in 2022 compared to 2021. There was a decrease in the searches for dyspnea (44.6 vs. 55.4; p<0.029) in Russia and for dizziness (87.6 vs. 92.8; p<0.005) worldwide. There was an increase in the searches for edema (93.6 vs. 91; p <0.002) and for fatigue (88.6 vs 79.5; p approaching 0) worldwide in study periods in 2022 compared to 2021 (Table 1).

**Table 1:** Trends in cardiac symptoms in 2022 when compared to 2021

	Russia			Ukraine			Worldwide		
Search terms for symptoms	Mean 2022	Mean 2021	p-value	Mean 2022	Mean 2021	p-value	Mean 2022	Mean 2021	p-value
Chest Pain	18.034	23.621	0.465	14.069	30.586	0.049	88.276	94.241	0.084
Dizziness	32.552	16.517	0.051	9.897	16.138	0.434	87.621	92.862	0.005
Palpitations	28.034	18.241	0.253	16.552	10.261	0.428	88.759	91.034	0.093
Shortness of Breath (Dyspnea)	44.655	55.483	0.029	51.931	60.483	0.168	89.793	91.828	0.093
Syncope	61.931	65.862	0.382	37.828	58.448	0.002	88.931	88.931	1.000
Cough	38.379	45.241	0.288	36.655	41.103	0.574	94.655	94.517	0.873
Heartburn	12.000	3.448	0.205	5.828	3.448	0.659	74.345	78.483	0.096
Fatigue	23.483	26.138	0.668	29.034	22.621	0.442	88.655	79.552	0.000
Leg Swelling (Pedal Edema)	51.517	56.380	0.355	40.069	66.414	0.000	93.621	91.000	0.002
Shoulder Pain	36.655	22.552	0.089	18.517	32.448	0.105	90.793	88.103	0.073
Nausea	25.759	16.670	0.168	12.724	21.517	0.321	90.931	92.310	0.303

There was no other significant difference between cardiac symptom search trends during the periods evaluated in Ukraine, Russia, and worldwide.

## Discussion

The possible explanations for a significant reduction in search for chest pain (14 vs. 30.5; p<0.049), pedal edema (40.0 vs 66.6; p approaching 0), and syncope (37.8 vs. 58.4; p<0.002) in Ukraine during the study periods in 2022 compared to 2021 are multifold. First, people tend to forego healthcare as a priority in a very stressful situation where their core existence is in jeopardy, during which survival, food, and shelter take precedence. Second, access to the Internet and technology may be disrupted due to the war. Third, the large-scale displacement of Ukrainians from their homes could have limited their access to electronic devices and Internet searches. Holman et al. (2008) demonstrated an increase in cardiovascular ailments like hypertension and heart problems at long-term follow-ups in a national sample after the 9/11 attacks [5]. Korinek et al. (2020) in a study of Vietnamese older adults showed a positive association between cardiovascular diseases and wartime exposure [6]. These implications for cardiovascular diseases might manifest later and might not be evident in Google Trends searches in this limited time. Also, anti-vaccine searches during the coronavirus pandemic have shown insights into health-seeking behavior despite the gravity of coronavirus disease 2019 (COVID-19)-related mortality [7]. Furthermore, real-time Google Trends is an inexpensive way to monitor the opioid overdose epidemic if correlated with the official records [8].

The limitation of the study is that the search terms may not translate into seeking health-related behavior. This has to be correlated with official health records to find the validity of searches into real-world translation. Disruption of Internet services in Ukraine may have caused fewer searches overall due to lack of availability. Another limitation of the study is that city or state-specific data are not available. As we know some cities in Ukraine were affected more than others. The strengths of the study are that it gives search results of related terms in all languages, so a language bias is eliminated. Google is a widely used search engine across the web, therefore the trends can be said to be quite comprehensive.

## Conclusions

In conclusion, there appears to be a significant reduction in searching for cardiovascular symptoms, namely, chest pain, pedal edema, and syncope in Ukraine two weeks before and after the war starting February 24, 2022, when compared to the same period in 2021. Given the limitations of the study, the reduction in searching may be due to a focus on other problems related to war and the availability of the Internet. Nonetheless, Google Trends provides us with meaningful insight into health-seeking behavior, which may help public health at large.
